# Lethal and Sublethal Effects of Selected Insecticides on the Eggs of the Predatory Bug *Orius niger*

**DOI:** 10.3390/insects17030346

**Published:** 2026-03-21

**Authors:** Isse Hassan Ali, Utku Yükselbaba

**Affiliations:** Department of Plant Protection, Faculty of Agriculture, Akdeniz University, Antalya 07059, Türkiye; bukhaariyare82@gmail.com

**Keywords:** bug, pesticide, thrips, biological control, life table

## Abstract

The compatibility of insecticides with biological control agents is a critical component of integrated pest management (IPM). In this study, the lethal and sublethal effects of acrinactrin, chlorantraniliprole, flupyradifurone, pyriproxyfen, spinosad, and spiromesifen on the egg stage of *Orius niger* (Wollf) (Hemiptera: Anthocoridae) were evaluated under laboratory conditions. Egg hatchability, immature survival, reproductive performance, and population parameters were analyzed using the age-stage, two-sex life table. Egg hatchability was lowest in the acrinactrin treatment (51%) and highest in the pyriproxyfen treatment (93%). Nymphal survival varied from 0% to 80%, with acrinactrin causing complete mortality and a significant reduction in spinosad, while the highest nymphal survival and population growth was recorded in spiromesifen treatment. The intrinsic rate of increase (*r*, day^−1^) was 0.00, 0.05, 0.05, 0.08, 0.004, and 0.06 for acrinactrin, chlorantraniliprole, flupyradifurone, pyriproxyfen, spinosad, and spiromesifen, respectively, while fecundity (*F*, eggs female^−1^) values were 0, 15.20, 15.83, 42.32, 10.37, and 21.85, respectively. According to the International Organization for Biological Control (IOBC) classification, acrinactrin was harmful, spinosad moderately harmful, and the remaining insecticides slightly harmful to *O. niger* eggs. Pyriproxyfen and spiromesifen were the most compatible with IPM programs. Caution is warranted for chlorantraniliprole due to its effects on reproductive parameters, whereas spinosad and acrinactrin should be avoided on *O. niger* eggs.

## Simple Summary

*Orius niger* is an important beneficial predator used to control insect pests in agricultural systems. However, chemical insecticides applied against pests may also harm this predator. In this study, we evaluated the effects of six commonly used insecticides on the egg stage of *O. niger*. We found that acrinactrin and spinosad caused strong negative effects on egg survival and later development, making them unsuitable for use where this predator is present. In contrast, pyriproxyfen, flupyradifurone, chlorantraniliprole and spiromesifen showed limited harmful effects and were more compatible with *O. niger*. These results provide useful information for selecting insecticides that can be safely integrated with biological control in integrated pest management programs.

## 1. Introduction

Biological control, the use of natural enemies to suppress pest populations, represents a cornerstone of integrated pest management (IPM) strategies worldwide [1]. Among these natural enemies, generalist predators of the genus “Orius” (Hemiptera: Anthocoridae), commonly known as minute pirate bugs, are critically important agents in agricultural ecosystems [2,3,4]. These polyphagous predators are highly effective against a wide range of small, soft-bodied arthropod pests, including thrips, spider mites, aphids, and lepidopteran eggs [4,5]. *Orius niger* (Wollf) (Hemiptera: Anthocoridae) is a key species native to and widely distributed across the Palearctic region, playing a significant role in the natural control of pests in both open field and protected cultivation systems, such as vegetables, ornamentals, and fruit orchards [6]. The success of IPM programs relies on the strategic and complementary use of multiple control tactics, with chemical control often remaining necessary for rapid pest suppression during outbreaks. However, the non-target effects of insecticides on beneficial arthropods, including predators and parasitoids, pose a significant threat to the stability and resilience of IPM systems [7,8]. Broad-spectrum conventional insecticides can cause severe direct mortality (lethal effects) in natural enemy populations, but perhaps more insidiously, they can also induce a range of sublethal effects [9]. These include reduced fecundity and fertility, impaired development, altered foraging behavior, and decreased predation capacity, which can ultimately disrupt biological control services and lead to secondary pest outbreaks [10,11].

In response to these challenges, the agricultural industry has increasingly shifted towards newer, more selective classes of insecticides, including butenolides (e.g., flupyradifurone), spinosyns (e.g., spinosad), insect growth regulators (IGRs) (e.g., pyriproxyfen), diamides (e.g., chlorantraniliprole), and tetronic acid derivatives (e.g., spiromesifen) [12,13]. These active ingredients are often promoted as more compatible with IPM because of their presumed lower toxicity to beneficial insects, as they frequently target specific physiological pathways that are less ubiquitous or less critical in natural enemies compared to pest species [14,15]. While the lethal effects of these newer chemistries on adult and nymphal stages of beneficial insects have been somewhat investigated, their impact on the egg stage, a vulnerable and often overlooked life stage, remains critically understudied [16]. The egg stage is immobile and cannot avoid exposure to pesticide residues on plant surfaces [17]. Exposure can occur through direct contact with spray deposits or through transovarial transmission from a contaminated parent, potentially leading to ovicidal effects or latent sublethal consequences that manifest later in development [18,19]. Assessing the impact on eggs is therefore essential for a complete and realistic risk assessment of any pesticide [20,21,22].

Consequently, this study aimed to determine the lethal and sublethal effects of six modern active ingredients—flupyradifurone, spinosad, pyriproxyfen, chlorantraniliprole, spiromesifen, and acrinathrin—on the egg stage of the predator *O. niger*. This study aims to provide a comprehensive toxicological profile by evaluating lethal and sublethal effects, including egg hatching rate, nymphal survival, developmental duration, and life table parameters. The findings will provide crucial data to guide the selection of insecticides that are compatible with conservation biological control strategies, thereby supporting the sustainability of agricultural production systems.

## 2. Materials and Methods

### 2.1. Insecticides

In this study, the active ingredients pyriproxyfen (Admiral^®^ 10 EC, 100 g/L, Sumitomo Chemical, Tokyo, Japan), spinosad (Laser^®^ SC, 480 g/L, Corteva Agriscience, Versoix, Switzerland), spiromesifen (Oberon^®^ SC, 240 g/L, Bayer CropScience, Leverkusen, Germany), acrinathrin (Rufast^®^ EW, 75 g/L, FMC Corporation, Harboøre, Denmark), flupyradifurone (Sivanto^®^ SL, 200 g/L, Bayer CropScience, Leverkusen, Germany), and chlorantraniliprole (Altacor^®^ WG, 35%, FMC Corporation, Philadelphia, PA, USA), which are commonly used against key pests such as *Bemisia tabaci*, *Frankliniella occidentalis*, and *Tuta absoluta,* were purchased as commercial formulations and used for the experiments (Table 1).

### 2.2. Insect Populations and Plant Material

*Orius niger* individuals were collected from Antalya and identified based on male morphology [23]. They were subsequently reared for at least three generations in plastic containers under controlled conditions (26 ± 1 °C, 60 ± 10% RH, 16:8 L:D), with *Ephestia kuehniella* eggs provided as food every two days [24]. Fresh pepper fruits were provided as oviposition substrates and replaced every two days. Fruits containing eggs were transferred to separate containers for development, with napkins placed inside to reduce cannibalism. For the experiments, cowpea (*Vigna unguiculata*) plants were cultivated in a peat–perlite substrate under the same controlled conditions described above, without insecticide application, and were subsequently used in the experiments.

### 2.3. Insecticide Bioassays

Experiments were conducted using cowpea leaf disks placed in plastic Petri dishes (3.5 cm diameter). Approximately 3 mL of 1.5% agar was poured into each dish and allowed to solidify for 2–3 min to prevent evaporation. Cowpea leaves, cut to the same diameter, were placed on the agar with the abaxial (lower) surface facing upward. One male and one female *O. niger*, identified from stock cultures based on ovipositor morphology, were transferred onto each leaf disk to allow oviposition. After 24 h, the leaf disks were inspected under a stereomicroscope, and the eggs laid were marked with a pen, with the oviposition date and time recorded. This bioassay protocol was adapted from previous studies [25], with modifications including the use of cowpea leaf disks for oviposition. Leaf disks containing *O. niger* eggs were then immersed for 10 s in 100 mL of the recommended field dose insecticide solution for each active ingredient and in distilled water for the control. At least 10 separate leaf disks (replicates) were used for each insecticide, with each disk containing a minimum of 7–8 eggs. The lethal effects of insecticides on *O. niger* eggs were determined by dividing the total number of eggs by the number of unhatched eggs.

### 2.4. Assessment of Biological Parameters of Orius niger

Leaf disks treated with insecticide solutions were maintained under controlled conditions of 26 ± 1 °C temperature, 60 ± 10% relative humidity, and a 16:8 h light: dark photoperiod. Labeled eggs were examined under a stereo microscope at 12 h intervals, and the hatching date and time of each egg were recorded. The egg incubation period was calculated by subtracting the oviposition date from the hatching date, while the hatching rate was calculated by subtracting the number of unhatched eggs from the total number of eggs. Eggs that did not hatch within 8 days were considered affected. Subsequent biological parameters were assessed based on the number of hatched eggs [25].

Newly emerged nymphs were immediately transferred to new insecticide-free leaf disks, with one nymph per disk. *E. kuehniella* eggs were then provided ad libitum, and the Petri dishes were sealed with plastic film. To ensure ventilation while preventing insect escape, small holes were made in the plastic wrap using fine No. 0 insect pins. Leaf disks were examined at 12 h intervals, and the date and time of each molt identified by the presence of exuviae were recorded to determine nymphal stage transitions. Each nymphal instar was monitored separately to determine stage-specific development time and survival until adulthood. The development time of the first nymphal instar was calculated as the interval between egg hatching and the first molt, while the development time of subsequent instars was determined as the interval between successive molts. Total development time was calculated from egg to adult emergence. Leaf disks were replaced every 2–3 days throughout the experiment. The nymph developmental stage at which death occurred was recorded. Stage-specific nymphal survival rates were calculated by subtracting the number of dead nymphs from the total number of hatched nymphs. Overall nymphal survival was determined based on the number of individuals reaching adulthood relative to the number of hatched eggs.

The total number of individuals that progressed from the nymph stage to the adult stage was noted, and their sex was determined by examining their ovipositor. The number of males and females was also noted. Following sex determination, adults were transferred onto leaf disks containing *E. kuehniella* eggs, arranged as one female and one male per leaf disk and were checked every 12 h. APOP (Adult Pre-Oviposition Period) was determined to be the time interval between adult emergence and the first oviposition, whereas TPOP (Total Pre-Oviposition Period) was calculated as the total time from the egg stage to the first oviposition, including both developmental and adult pre-reproductive periods. The time from adult emergence to death was recorded to determine the longevity of female and male individuals separately. Cowpea leaf disks used in the experiments were replaced every 2–3 days.

### 2.5. Data and Life Table Analysis

Life table data and related biological parameters were analyzed using the age-stage, two-sex life table method implemented in the TWOSEX-MSChart program (v. 10.1.2024) [26]. Biological parameters of *O. niger*, including the age-stage-specific survival rate (*s_x__j_*), which represents the probability that a newborn individual survives to age *x* and stage *j* (where *x* and *j* represent age and developmental stage, respectively); the age-specific survival rate (*l_x_*) and age-specific fecundity (*m_x_*); life expectancy (*e_x__j_*), which indicates the average remaining lifespan of individuals at age *x* and stage *j*; and the age-stage-specific reproductive value (*v_x__j_*), which represents the contribution of individuals at age *x* and stage *j* to future generations, were analyzed [26]. In addition, life cycle parameters such as the intrinsic rate of increase (*r*), net reproductive rate (*R*_0_), mean generation time (*T*), finite rate of increase (*λ*), and population doubling time (*DT*) were analyzed according to the age and stage-specific twosex life table using the TWOSEX-MSChart program and the differences in these parameters of *O. niger* among insecticides were analyzed using the paired Bootstrap test method (m = 1,000,000) [26,27]. The effects of different insecticide treatments on egg hatching and developmental parameters of *O. niger* were analyzed using one-way analysis of variance (ANOVA). When significant differences were detected, Duncan’s multiple range test was applied to separate means at (*p* ≤ 0.05).

The age–stage specific survival rate (*l_x_*) and the age-specific fecundity (*m_x_*) were calculated according to Formulas (1) and (2) [26]

(1) $$Ix=\sum_{\dot{j}=1}^{\beta }s\cdot x_{j}$$

(2) $$m_{x}=\frac{\sum_{J=1}^{\beta }s_{x_{i}}F_{xj}}{\sum_{j=1}^{\beta }s_{xj}}$$

The age–stage specific life expectancy (*e_xj_*) was calculated using Formula (3) [27]

(3) $$exj=\sum_{i=x}^{\infty }\sum_{y=j}^{I}S_{iy}^{\prime }$$

The reproductive value (*v_x__j_*) was calculated assuming *s*′ = 1 using Formula (4) [28]

(4) $$v_{xj}=\frac{e^{\Gamma \left(x+1\right)}}{s_{xj}}\sum_{i=x}^{\infty }e^{-\Gamma \left(i+1\right)}\sum_{y=j}^{\beta }S_{iy}f_{iy}$$

The intrinsic rate of increase (*r*) was calculated according to Formula (5) [29]

(5) $$r=\sum_{x=0}^{\infty }e^{-r\left(x+1\right)}I_{x}m_{x}=1$$

The net reproductive rate (*R*_0_) was calculated according to Formula (6) [30]

*R*_0_ = Σ *lxmx* (6)

The finite rate of increase (*λ*) was calculated according to Formula (7) [29].

*λ* = *e^y^* (7)

The mean generation time (*T*) was calculated according to Formula (8) [31]

*T* = *LnR*_0_/*r* (8)

### 2.6. Toxicity Classification Criteria

Insecticides were classified according to the criteria established by the IOBC Working Group “Pesticides and Beneficial Organisms” (https://iobc-wprs.org/, accessed on 14 September 2025). Mortality observed in treated groups was corrected relative to the control using Abbott’s formula [32]. The total effect (*E*) of each insecticide, integrating both lethal and sublethal effects, was calculated following the formula [33].

$$E(\%)=100-(100-M_{c})\times E\pm R$$

where *M_c_* represents the final corrected mortality rate, and ER denotes the effect on oviposition, calculated as $ER=R_{t}/R_{c}$, with $R_{t}$ and $R_{c}$ being the mean numbers of ovipositions in the treated and control groups, respectively. Insecticides were assigned to IOBC toxicity classes based on E values as: class 1 (harmless), *E* < 30%; class 2 (slightly harmful), 30% ≤ *E* < 80%; class 3 (moderately harmful), 80% ≤ *E* < 99% and Class 4 (harmful), *E* ≥ 99% [33].

## 3. Results

### 3.1. Lethal Effects of Insecticides on Orius niger Eggs

The lethal effects of insecticides on *O. niger* eggs were shown in Table 2. Egg mortality due to hatching failure was 49% for acrinathrin, 7% for pyriproxyfen, and 17%, 18%, and 19% for flupyradifurone, spiromesifen, and spinosad, respectively, while it was only 4% in the control treatment. Significant differences in lethal effects were observed between the control and the acrinathrin, flupyradifurone, spinosad, and spiromesifen treatments. In contrast, no statistically significant difference was detected between the chlorantraniliprole and pyriproxyfen treatments and the control (Table 2).

### 3.2. Biological Parameters of Orius niger

The biological parameters of *O. niger* following insecticide bioassays on the eggs are presented in Table 2. The mean egg hatching time was 4.17 days in the control group. Acrinathrin treatment resulted in the longest hatching at 4.30 days, whereas flupyradifurone and pyriproxyfen showed the shortest hatching at 3.90 days. Similarly, mean egg hatching times of 4.1 days were recorded for the chlorantraniliprole and spiromesifen treatments, whereas spinosad had a mean of 4.0 days. Compared with the control, chlorantraniliprole and spiromesifen did not differ significantly, while acrinathrin differed significantly from all other treatments. The total nymphal development time of flupyradifurone, spiromesifen, chlorantraniliprole, spinosad and pyriproxyfen treatments was determined as 11.8, 12.3, 13.7, 13, and 12.6 days, respectively and 12.6 days in the control. The longest development time was observed in the chlorantraniliprole treatment (13.7 days), which differed significantly from the control and all other insecticides. In contrast, the shortest nymphal development time was recorded with flupyradifurone 11.8 (days), which was also significantly different from the control and other treatments. Nymphal development times in the pyriproxyfen, spiromesifen, and spinosad treatments did not differ significantly from the control. No individuals reached adulthood in the acrinathrin treatment (Table 2).

Nymphal survival rates were 65%, 80%, 75%, 28%, and 61% for flupyradifurone, spiromesifen, chlorantraniliprole, spinosad, and pyriproxyfen treatments, respectively, and 82% in the control group. Survival was highest in the control group and varied among the insecticide treatments, with the lowest survival observed in the spinosad treatment and the highest in the spiromesifen treatment. No survival rate was determined in the acrinathrin group as no individual reached adulthood. Survival rates in the chlorantraniliprole and spiromesifen treatments did not differ significantly from the control, whereas significant reductions were observed in the flupyradifurone and pyriproxyfen (Table 2). The Spinosad treatment group was significantly different from all other treatments and the control group. The adult pre-oviposition period (APOP) was 7.0 days in the control group and 6.84, 6.92, 10.6, 7.7, and 5.84 days in flupyradifurone, spiromesifen, chlorantraniliprole, spinosad, and pyriproxyfen, respectively, with chlorantraniliprole having the highest APOP and no significant differences between the control and all other insecticide treatments. In contrast, the total pre-oviposition period (TPOP) of flupyradifurone, spiromesifen, chlorantraniliprole, spinosad and pyriproxyfen was determined as 22.8, 23.2, 28.5, 24.2, 22.13, respectively and 23.5 in the control group. The chlorantraniliprole group had the highest TPOP (28.5 days), which differed significantly from the control and all other treatments, except spinosad. Among other treatments and the control group, the TPOP value did not differ significantly from one another. Female longevity was determined as 16.7, 25.2, 21.7, 14.6, 15.8 days in flupyradifurone, spiromesifen, chlorantraniliprole, spinosad and pyriproxyfen, respectively, with spinosad having the lowest (14.6 days) and spiromesifen having the highest (25.2 days). The mean female lifespan in the control group was 19.6 days with no significant differences between the control and insecticide treatments; however, female longevity in the spiromesifen treatment differed significantly from that in the flupyradifurone, pyriproxyfen, and spinosad treatments, but not from chlorantraniliprole (Table 2). In contrast, the male lifespan was 10.19, 15.2, 17.1, 10.4, 12.3 days in flupyradifurone, spiromesifen, chlorantraniliprole, spinosad, pyriproxyfen, respectively and 14 days in the control group, with no significant difference between all treatments and the control group. The fecundity rate (number of oviposited females divided by the total number of females in a group) was 38%, 35%, 35%, 38%, 76% in flupyradifurone, spiromesifen, chlorantraniliprole, spinosad, and pyriproxyfen, respectively and 64% in the control group. While pyriproxyfen differed significantly from the control group and all treatments, there was no significant difference between all treatments and the control group.

### 3.3. Life Table Analyses

Population parameters of *O. niger* following insecticide exposure at the egg stage were calculated separately for each active ingredient using the TWOSEX-MSChart program, which accounts for both sexes in population parameter estimation [26]. The intrinsic rate of increase (*r*), finite rate of increase (*λ*), net reproductive rate (*R*_0_), mean generation time (*T*), fecundity (*F*) and population doubling time (*DT*) for each treatment are presented in Table 3.

Population growth parameters were highest in control and pyriproxyfen treatments, while spiromesifen caused moderate reductions. Flupyradifurone and chlorantraniliprole significantly suppressed population growth by reducing *r*, *R*_0_, and *F* and prolonging *T*. Spinosad had the strongest negative effect on all parameters. No estimates were obtained for acrinathrin due to complete mortality.

**Figure 1:**
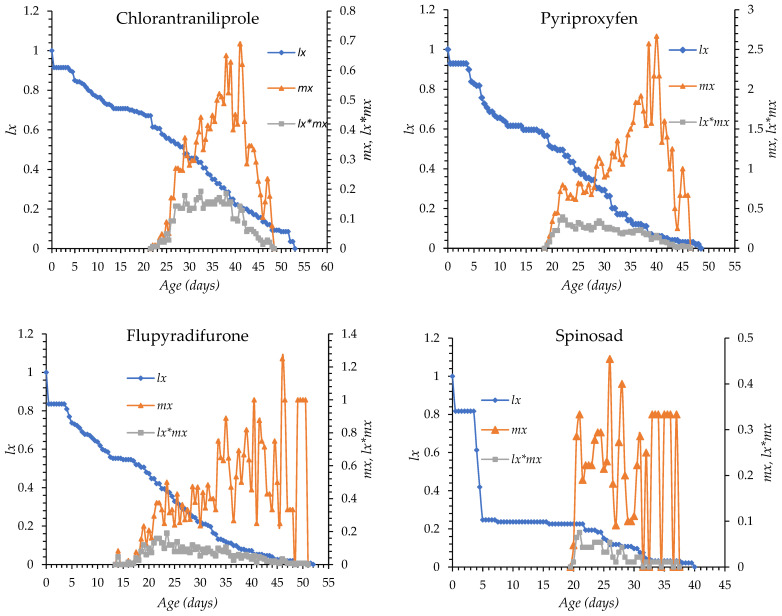

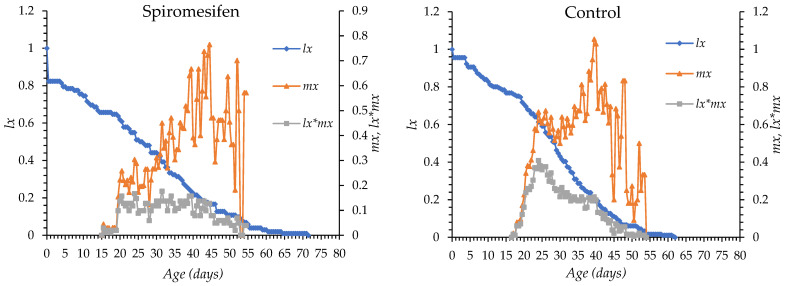
*l_x_*: Age-specific survival, *m_x_*: fecundity curves of *Orius niger* for all insecticide treatments and control.

The age-specific survival (*l_x_*) and fecundity (*m_x_*) curves of *O. niger* exposed to chlorantraniliprole, pyriproxyfen, flupyradifurone, spinosad, spiromesifen, and control treatment are shown in Figure 1. In all treatments, mortality began between days 4 and 5 after egg hatching in all groups. Survival declined gradually with chlorantraniliprole, pyriproxyfen, flupyradifurone, and spiromesifen, whereas a more rapid decline was observed with spinosad. The longest survival period occurred under spiromesifen (up to day 72) and in control (day 63), while survival ended earlier under the other insecticides. The onset of oviposition was day 14 in the flupyradifurone, day 22 in the chlorantraniliprole, day 18 in pyriproxyfen, day 19 in spinosad, day 15 in spiromesifen and day 16 in the control group. Peak fecundity occurred between days 26 and 46, with the earliest peak observed in the spinosad treatment and the latest in the flupyradifurone treatment. Overall, insecticide treatments altered the timing and intensity of survival and reproductive patterns compared with the control (Figure 1).

**Figure 2:**
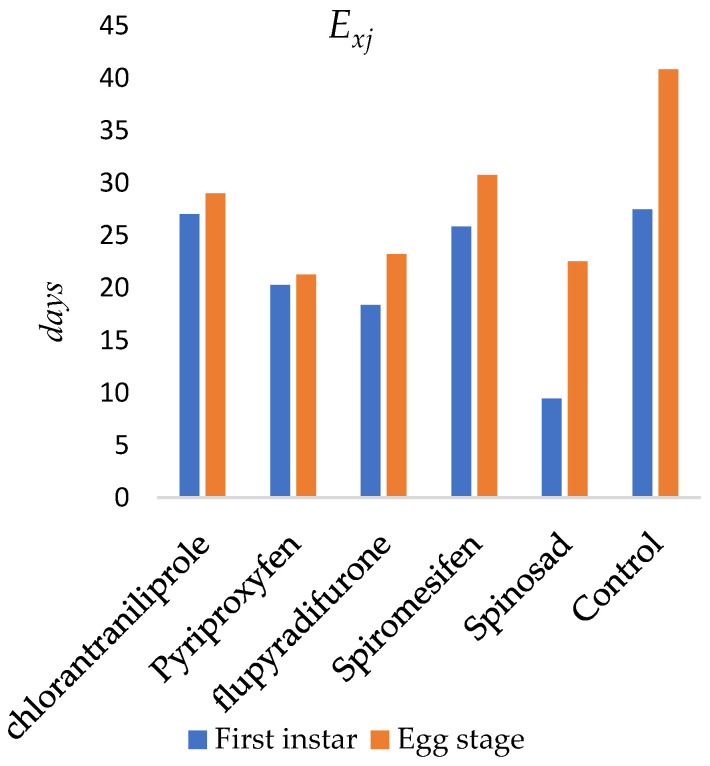
Life expectancy (*e_xj_*) of newly emerged *Orius niger*.

The age-specific life expectancy (*e_xj_*) of *O. niger* following insecticide applications to the egg stage is presented in Figure 2. The expected lifespan of newly emerged individuals was estimated as 27.04 days for chlorantraniliprole, 20.26 days for pyriproxyfen, 18.35 days for flupyradifurone, 9.44 days for spinosad, and 25.84 days for spiromesifen. In the control group, the expected lifespan was 27.46 days. The maximum life expectancy occurred at the egg stage (day 1) and was estimated as 29.02 days for chlorantraniliprole, 21.26 days for pyriproxyfen, 23.22 days for flupyradifurone, 22.50 days for spinosad, 30.77 days for spiromesifen, and 40.85 days in the control group.

The age-stage and sex-specific survival rates (*s_xj_*) across all treatments showed that mortality during the immature stages was most pronounced in the third to fifth nymphal instars. Under chlorantraniliprole and pyriproxyfen exposure, survival remained relatively high during the egg and early nymphal stages but declined markedly at the fourth nymphal stage.

Adult survival rate in chlorantraniliprole was similar for females and males (32%), but in pyriproxyfen, the adult female and male survival rate was 29% and 32%, respectively. Flupyradifurone caused a gradual reduction in survival from early developmental stages, with the highest mortality observed during the third nymphal stage, resulting in adult survival rates of 24% for females and 25% for males. In contrast, spinosad produced the lowest survival rates across all developmental stages, particularly during the early nymphal instars, and resulted in markedly reduced adult survival (9% for females and 14% for males). Spiromesifen and the control treatment exhibited comparatively higher survival throughout development, although increased mortality was observed during the fourth and fifth nymphal stages. Adult survival rate in spiromesifen was 32% for females and 30% for males, and in the control group, this value was 38% for females and 31% for males.

In the age–stage specific reproductive value (*v_xj_*), the highest reproductive value of *O. niger* was observed at day 27 (12.63) for chlorantraniliprole, at days 21, 31, and 32 (25) for pyriproxyfen, at day 21 (11.33) for flupyradifurone, and at day 17 (10.70) for spinosad. In the spiromesifen treatment, peak reproductive values occurred during the adult stage at days 24 (11.34) and 29 (11.27). The control group showed a maximum *vxj* of 16 on day 23.

### 3.4. Toxicity Classification

The IOBC-based toxicity classification of the tested insecticides on *O. niger* eggs, calculated using the total effect (*E*) according to the formula described in Section 2.6 and following IOBC criteria, is summarized in Table 4. Acrinactrin exhibited the highest toxicity and was classified as harmful (Class 4). Spinosad was categorized as moderately harmful (Class 3). In contrast, flupyradifurone, spiromesifen, pyriproxyfen and chlorantraniliprole were assigned to Class 2 and considered slightly harmful to *O. niger* eggs (Table 4).

## 4. Discussion

Species belonging to the family Anthocoridae are recognized as important agents in biological control programs [34]. *Orius* species are widely and effectively used for the control of thrips, particularly in protected cultivation systems [35]. *Orius niger* is a polyphagous species widely distributed throughout the Western Palearctic region [35]. In integrated pest management (IPM) programs, the compatibility between insecticides and natural enemies is a critical factor for effective pest management [36,37]. Evaluating the effects of pesticides on life table parameters is considered one of the most comprehensive approaches for assessing both lethal and sublethal effects [37,38]. Life table studies, including assessments of demographic parameters, provide valuable information on the impacts of insecticides on natural enemies and support their integration into IPM programs [7]. In this study, the lethal effects of insecticides on the egg stage of *O. niger* and the sublethal effects on life table parameters were investigated. Egg hatching rates for the control, acrinactrin, chlorantraniliprole, flupyradifurone, pyriproxyfen, spinosad, and spiromesifen treatments were 96%, 51%, 91%, 83%, 93%, 81%, and 82%, respectively. While flupyradifurone, spinosad, and spiromesifen exhibited ovicidal activity and reduced egg hatching rates, acrinactrin showed a pronounced inhibitory effect on egg hatching in *O. niger*. In contrast, chlorantraniliprole and pyriproxyfen did not adversely affect egg hatching. Consistent with our findings, 72% egg hatching rate was reported when spinosad was applied to *Orius majusculus* eggs, indicating a reduction compared with the control [39]. The toxicity of pymetrozine, rynaxypyr and pyriproxyfen on *Orius insidiosus* eggs did not exhibit ovicidal activity [25]. In contrast, abamectin, cartap hydrochloride, spirotetramat + imidacloprid, and flubendiamide were reported to have ovicidal effects to varying degrees [25]. The lack of ovicidal activity of pymetrozine, rynaxypyr and pyriproxyfen may be attributed to their high molecular weights, which likely limit their penetration into the egg and thus prevent ovicidal effects [25]. On the other hand, they also reported that the reduction in viability of *O. insidiosus* eggs caused by abamectin, spirotetramat + imidacloprid, and flubendiamide may result from their high octanol–water partition coefficient (log Kow) values [25]. In this study, the egg hatching period was not significantly affected by most active ingredients, suggesting limited interference with embryonic development. However, acrinactrin exhibited a negative effect, indicating higher ovicidal or embryotoxic potential than the other compounds tested. Compared with the control, chlorantraniliprole and spiromesifen did not alter egg hatching period, whereas shorter hatching periods were observed for flupyradifurone, pyriproxyfen, and spinosad. The absence of changes in egg hatching period following chlorantraniliprole and spiromesifen exposure may be attributed to their specific modes of action. Chlorantraniliprole primarily targets muscle calcium channels, whereas spiromesifen affects lipid biosynthesis, and both pathways are relatively inactive during early embryogenesis. In contrast, the shortened egg hatching period observed for flupyradifurone, pyriproxyfen, and spinosad may result from neurotoxic or endocrine-mediated stress responses, potentially accelerating embryonic development as an adaptive response. Similarly, abamectin and spirotetramat + imidacloprid affected the egg hatching duration of *O. insidiosus* but did not affect the other active ingredients [25]. Nymphal survival rates for the control, acrinactrin, chlorantraniliprole, flupyradifurone, pyriproxyfen, spinosad, and spiromesifen treatments were 82%, 0%, 75%, 65%, 61%, 28%, and 80%, respectively. Sublethal effects on nymphal survival were observed for flupyradifurone, pyriproxyfen, spinosad, and spiromesifen, whereas no nymphs reached adulthood in the acrinactrin treatment. Chlorantraniliprole and spiromesifen had survival rates comparable to the control, suggesting no sublethal effects. The marked reduction in nymphal survival observed with acrinactrin and spinosad is likely attributable to their neurotoxic modes of action, which disrupt sodium channel function and nicotinic/GABAergic neurotransmission, respectively. In contrast, pyriproxyfen reduced survival presumably through endocrine-mediated disruption of molting processes, while flupyradifurone induced moderate mortality consistent with its activity on nicotinic acetylcholine receptors. Conversely, chlorantraniliprole and spiromesifen exhibited survival rates comparable to the control, suggesting greater physiological selectivity and compatibility with *Orius* spp., possibly due to lower receptor sensitivity or reduced impact on essential metabolic pathways during nymphal development. In agreement with our findings, the acute toxicity of adults of *O. strigicollis* was highly susceptible to flupyradifurone, whereas immature stages exhibited significant sublethal and developmental effects, suggesting stage-specific sensitivity to this neuroactive insecticide [40]. Although there are no significant sublethal effects of pyriproxyfen on nymphal survival of *O. insidiosus* [25], the reduced survival observed in the present study may reflect species differences and species -specific differences in juvenile hormone sensitivity, variations in exposure concentration or duration, and differences in developmental stage susceptibility. As pyriproxyfen acts as a juvenile hormone analog, even subtle differences in endocrine regulation may result in divergent developmental outcomes among closely related *Orius* species.

Chlorantraniliprole exhibited sublethal effects by prolonging total nymphal development time, whereas the shortest nymphal development period was observed following flupyradifurone exposure. No adverse effects on total nymphal development time were observed for the other active substances compared with the control. The effects of pyriproxyfen were consistent between the present study and [25], with no significant impact on nymphal development time detected. In this study, the egg hatching period was generally not affected by most active ingredients, indicating limited interference with embryonic development. Acrinactrin, however, exhibited strong ovicidal potential, while flupyradifurone, pyriproxyfen, and spinosad caused shorter egg hatching periods, likely due to neurotoxic or endocrine-mediated stress responses. Chlorantraniliprole and spiromesifen did not alter egg hatching, consistent with their modes of action targeting muscle calcium channels and lipid biosynthesis, respectively, which are relatively inactive during early embryogenesis. Nymphal survival rates mirrored these patterns: acrinactrin and spinosad, both neuroactive compounds, caused high mortality, pyriproxyfen reduced survival through endocrine disruption, and flupyradifurone induced moderate mortality via nicotinic acetylcholine receptor activity, whereas chlorantraniliprole and spiromesifen showed minimal sublethal effects, suggesting physiological selectivity. Linking these findings to IRAC classifications highlights the applied relevance, as considering modes of action when selecting and rotating insecticides can help minimize unintended impacts on beneficial predators and support integrated pest management strategies.

Female longevity following bioassays with the control, chlorantraniliprole, flupyradifurone, pyriproxyfen, spinosad, and spiromesifen was recorded as 19.6, 21.7, 16.7, 15.8, 14.6, and 25.2 days, respectively. The shortest female lifespans were observed under flupyradifurone, pyriproxyfen, and spinosad treatments; however, these reductions were not statistically significant compared with the control and therefore did not indicate sublethal effects on female longevity. The proportion of ovipositing females was recorded as 35%, 38%, 38%, and 35% for chlorantraniliprole, flupyradifurone, spinosad, and spiromesifen, respectively, whereas higher proportions were observed in the control (64%) and pyriproxyfen (76%) treatments. The reduced proportion of ovipositing females observed in the chlorantraniliprole, flupyradifurone, spinosad, and spiromesifen treatments may reflect sublethal physiological stress affecting feeding, energy allocation, calcium signaling, or lipid metabolism. In contrast, the higher oviposition rate recorded in the pyriproxyfen treatment is likely attributable to its role as a juvenile hormone analog, which may stimulate ovarian development and reproductive activity at sublethal concentrations. According to toxicity classification, chlorantraniliprole, flupyradifurone, pyriproxyfen, and spiromesifen were categorized as slightly harmful, spinosad as moderately harmful, and acrinactrin as harmful. Life table analysis revealed that the net reproductive rate (*R*_0_) did not differ significantly among the control, pyriproxyfen, and spiromesifen treatments. Similarly, fecundity values for the control, pyriproxyfen, and spiromesifen were 30.28, 42.32, and 21.85, respectively, with no significant differences among them. The similarity in *R*_0_ among the control, pyriproxyfen, and spiromesifen treatments suggests that these compounds did not significantly impair survival or reproductive output. In contrast, the other insecticides likely reduced population growth through combined effects on survival, development, and age-specific fecundity, thereby lowering overall demographic performance. However, *R*_0_ was significantly lower under chlorantraniliprole exposure compared with these treatments and the control, resulting in a significantly lower fecundity value (15.20) compared with the control. Notably, flupyradifurone and spinosad produced markedly lower *R*_0_ and *F* values, indicating pronounced effects on population parameters. No life table parameters could be calculated for acrinactrin because no individuals reached the adult stage. Although chlorantraniliprole did not affect egg hatching rate, egg incubation duration, or nymphal survival, its sublethal effects were evident through prolonged total nymphal development time and a reduced proportion of ovipositing females, ultimately leading to decreased fecundity and negative effects on reproduction.

## 5. Conclusions

When the lethal effects of insecticides on egg hatching and their sublethal effects on developmental and population parameters are considered together, pyriproxyfen and spiromesifen exhibited only limited negative effects *on O. niger*. While chlorantraniliprole exhibited minimal negative effects on egg hatching and nymphal stages, its impact on reproductive parameters was lower than that of the control and should therefore be considered with caution. Overall, pyriproxyfen and spiromesifen can be recommended for use in IPM strategies targeting the egg stage of *O. niger*. Chlorantraniliprole may also be considered compatible with *O. niger*; however, its effects on reproductive parameters should be considered. Although flupyradifurone is classified as slightly harmful to *O. niger* eggs, its lethal effects on egg hatching and sublethal effects on biological parameters suggest that its use should be carefully evaluated in areas where biological control with *O. niger* is implemented. In contrast, spinosad and acrinactrin are not recommended for application at the egg stage in biological control programs involving *O. niger*. Further studies examining the lethal and sublethal effects of these active substances on the nymphal stages of *O. niger* would provide valuable information for optimizing chemical control strategies compatible with biological control programs.

## Figures and Tables

**Table 1:** Insecticides and their modes of action according to the IRAC classification.

Insecticide	IRAC Group	Mode of Action
Flupyradifurone	4D	Nicotinic acetylcholine receptor competitive modulator
Spinosad	5	Nicotinic acetylcholine receptor allosteric activator
Pyriproxyfen	7C	Juvenile hormone analog (insect growth regulator)
Chlorantraniliprole	28	Ryanodine receptor modulator
Spiromesifen	23	Acetyl-CoA carboxylase inhibitor (lipid biosynthesis inhibitor)
Acrinathrin	3A	Voltage-gated sodium channel modulator (pyrethroid)

IRAC: Insecticide Resistance Action Committee classification.

**Table 2:** Biological parameters of *Orius niger* following insecticide exposure at the egg stage.

Stage	Control		Flupyradifurone		Spiromesifen		Chlorantraniliprole		Acrinathrin		Spinosad		Pyriproxyfen		*p*	*df*	*F*
	N	Mean ± SE	N	Mean ± SE	N	Mean ± SE	N	Mean ± SE	N	Mean ± SE	N	Mean ± SE	N	Mean ± SE			
Egg hatching rate (%)	181	96 ± 0.03 ^a^	152	83 ± 0.03 ^bc^	102	82 ± 0.03 ^c^	142	91 ± 0.29 ^ab^	112	51 ± 0.03 ^d^	92	81 ± 0.04 ^c^	98	93 ± 0.04 ^a^	0.000	6	23.7
Egg hatching time (days)	174	4.17 ± 0.04 ^b^	127	3.9 ± 0.04 ^c^	84	4.1 ± 0.05 ^b^	129	4.1 ± 0.04 ^b^	57	4.3 ± 0.06 ^a^	76	4 ± 0.05 ^c^	91	3.9 ± 0.05 ^c^	0.000	6	11.86
Total nymph duration (days)	141	12.6 ± 0.1 ^bc^	83	11.8 ± 0.1 ^d^	67	12.3 ± 0.2 ^c^	98	13.7 ± 0.1 ^a^	Nd	Nd	21	13 ± 0.3 ^b^	57	12.6 ± 0.2 ^bc^	0.000	5	22.8
Nymph survival rate (%)	174	82 ± 0.02 ^a^	127	65 ± 0.04 ^bc^	84	80 ± 0.05 ^a^	130	75 ± 0.04 ^ab^	57	0	75	28 ± 0.05 ^d^	91	61 ± 0.04 ^c^	0.000	6	39.86
APOP (day)	47	7 ± 0.66 ^ab^	16	6.84 ± 1.13 ^ab^	12	6.92 ± 1.3 ^ab^	19	10.6 ± 1.04 ^a^	Nd	Nd	3	7.2 ± 2.6 ^ab^	19	5.84 ± 1.04 ^b^	0.037	5	2.46
TPOP (day)	47	23.5 ± 0.7 ^b^	16	22.8 ± 1.2 ^b^	12	23.2 ± 1.42 ^b^	19	28.5 ± 1.13 ^a^	Nd	Nd	3	24.2 ± 2.8 ^ab^	19	22.13 ± 1.1 ^b^	0.002	5	4.1
Female life expectancy (days)	73	19.6 ± 1.3 ^ab^	42	16.7 ± 1.7 ^b^	34	25.2 ± 1.9 ^a^	49	21.7 ± 1.6 ^ab^	Nd	Nd	8	14.6 ± 4 ^b^	25	15.8 ± 2.2 ^b^	0.005	5	3.49
Male life expectancy (days)	67	14 ± 1 ^abc^	41	10.19 ± 1.2 ^c^	33	15.2 ± 1.4 ^ab^	49	17.1 ± 1.1 ^a^	Nd	Nd	13	10.4 ± 2.2 ^c^	32	12.3 ± 1.4 ^bc^	0.001	5	4.46
Female ratio (%)	141	51.8 ± 0.04 ^a^	83	50.6 ± 0.05 ^a^	67	50.7 ± 0.06 ^a^	98	50 ± 0.05 ^a^	Nd	Nd	21	38 ± 0.1 ^a^	57	44 ± 0.06 ^a^	0.823	5	0.44
Fecundity rate (%)	73	64 ± 0.06 ^ab^	42	38 ± 0.07 ^b^	34	35 ± 0.08 ^b^	49	35 ± 0.07 ^b^	Nd	Nd	8	38 ± 0.17 ^b^	25	76 ± 0.1 ^a^	0.000	5	5.02

N, number of individuals used in bioassay; *df*: degrees of freedom; *F*: F-statistic; APOP: adult pre-oviposition period; TPOP: total pre-oviposition period; Nd: not determined; Means in the same row followed by the same letter are not statistically different (*p* > 0.05).

**Table 3:** Life table parameters of *Orius niger* following insecticide application at the egg stage.

Parameters	Estimates ± SE						
	Control	Spiromesifen	Pyriproxyfen	Flupyradifurone	Chlorantraniliprole	Acrinathrin	Spinosad
*r* (day^−1^)	0.09 ^a^	0.0 6^ab^	0.08 ^a^	0.05 ^b^	0.05 ^b^	Nd	0.004 ^d^
*λ* (day^−1^)	1.09 ^a^	1.06 ^ab^	1.09 ^a^	1.05 ^b^	1.05 ^b^	Nd	0.99 ^c^
*R*_0_ (offspring/female)	12.22 ^a^	7.28 ^ab^	10.69 ^ab^	4.38 ^bc^	5.32 ^b^	Nd	0.89 ^c^
*T* (days)	28.80 ^b^	31.86 ^a^	28.59 ^b^	27.79 ^b^	34.33 ^a^	Nd	25.29 ^ab^
*F* (eggs/female)	30.28 ^a^	21.85 ^ab^	42.32 ^a^	15.83 ^b^	15.20 ^b^	Nd	10.37 ^c^
*DT* (days)	7.98^b^	11.12 ^ab^	8.36 ^b^	13.04 ^a^	14.23 ^a^	Nd	Nd

Nd: Not determined; Means in the same row followed by the same letter are not statistically different (*p* > 0.05).

**Table 4:** Toxicity of active ingredients on *Orius niger* eggs.

Active Ingredient	*E* Value (%)	Toxicity Class	Status
Chlorantraniliprole	34.58	2	Slightly harmful
Pyriproxyfen	42.67	2	Slightly harmful
Spiromesifen	43.89	2	Slightly harmful
Flupyradifurone	52.78	2	Slightly harmful
Spinosad	80.74	3	Moderately harmful
Acrinathrin	100	4	Harmful

## Data Availability

The original contributions presented in this study are included in the article. Further inquiries can be directed to the corresponding author.
